# Magnoflorine—Isolation and the Anticancer Potential against NCI-H1299 Lung, MDA-MB-468 Breast, T98G Glioma, and TE671 Rhabdomyosarcoma Cancer Cells

**DOI:** 10.3390/biom10111532

**Published:** 2020-11-10

**Authors:** Estera Okon, Wirginia Kukula-Koch, Marta Halasa, Agata Jarzab, Marzena Baran, Magdalena Dmoszynska-Graniczka, Apostolis Angelis, Eleftherios Kalpoutzakis, Malgorzata Guz, Andrzej Stepulak, Anna Wawruszak

**Affiliations:** 1 Department of Biochemistry and Molecular Biology, Medical University of Lublin, 20-093 Lublin, Poland; estera.okon@umlub.pl (E.O.); marta.halasa@umlub.pl (M.H.); agata.jarzab@umlub.pl (A.J.); marzena.baran@umlub.pl (M.B.); magdalena.dmoszynska-graniczka@umlub.pl (M.D.-G.); malgorzata.guz@umlub.pl (M.G.); andrzej.stepulak@umlub.pl (A.S.); 2 Department of Pharmacognosy, Medical University of Lublin, 20-093 Lublin, Poland; 3 Laboratory of Pharmacognosy and Natural Products Chemistry, School of Pharmacy, National and Kapodistrian University of Athens, Panepistimioupoli Zografou, 15771 Athens, Greece; aangjel@pharm.uoa.gr (A.A.); elkalp@pharm.uoa.gr (E.K.)

**Keywords:** magnoflorine, natural products, isoquinoline alkaloids, anti-cancer activity, *Berberis cretica*, Berberidaceae, counter-current chromatography, CPC, HPLC-MS

## Abstract

Magnoflorine (MGN) is a quaternary aporphine alkaloid that exhibits numerous therapeutic properties, including neuropsychopharmacological, anti-anxiety, immunomodulatory, anti-inflammatory, antioxidant, or antifungal activities. The aim of the present study was an investigation of the influence of MGN on viability, proliferation, induction of apoptosis, and cell cycle arrest in NCI-H1299 lung, MDA-MB-468 breast, T98G glioma, and TE671 rhabdomyosarcoma cancer cells. MGN was isolated from the roots of *Berberis cretica* L. by counter-current partition chromatography (CPC). Cell viability and proliferation assessments were performed by means of MTT (3-(4,5-dimethylthiazol-2-yl)-2,5-diphenyltetrazolium bromide) and 5-bromo-2ʹ-deoxyuridine (BrDU) assays, respectively. The induction of apoptosis and cell cycle progression was measured using fluorescence-activated cell sorting analysis. MGN in high doses inhibits proliferation, induces apoptosis, and inhibits cell cycle in S/G2 phases in a dose-dependent manner. MGN seems to be a promising anti-cancer compound in therapy of some types of lung, breast, glioma, and rhabdomyosarcoma cancers, for which current standard therapies are limited or have severe strong side effects.

## 1. Introduction

Magnoflorine (MGN) is a quaternary aporphine alkaloid widely distributed among different representatives of Magnoliaceae, Menispermaceae, Berberidaceae, or Papaveraceae species [1]. It is clear that the recent decade of scientific research brings interest back to this compound. For the moment, there are already 45 publications containing a reference to MGN in the Scopus database solely in 2020. They include both the identification studies on MGN-containing plants and pharmacological evaluation of plant extracts rich in this alkaloid or MGN itself.

The published scientific papers list its vast therapeutic properties including: neuropsychopharmacological [2], anti-anxiety [3,4], immunomodulatory [5], anti-inflammatory [3], antioxidant [6] or antifungal [7] properties. Several studies evidenced also the anti-cancer activity of MGN [1,8,9]. Nevertheless, the mechanism of action and the influence of MGN on the progression of cancer that is of particular interest to the authors have not yet been fully understood [8,10].

It is worth emphasizing that despite an increasing number of reports on the biological activity of MGN, the recovery of this compound from the plant matrix on a preparative or industrial scale is still troublesome. High prices and weak availability of a reference compound restrict the research on this alkaloid. Based on these observations, there is a need to develop new isolation strategies that would provide pure MGN for further research.

Having studied a variety of plant extracts from the representatives of *Berberis*, *Argemone*, *Papaver*, and *Eschscholzia* genders [11], the authors selected the roots from *Berberis cretica* L. as the most rich source of MGN. Additionally, the composition of the extract seemed quite poor in other metabolites, and that is why this plant source seems to be a promising one for the future isolation of this aporphine alkaloid for pharmacological treatment.

In this work, the authors implemented counter-current partition chromatography to isolate MGN from the roots of Cretan barberry (*Berberis cretica*). This particular separation technique allows for the complete elimination of a solid adsorbent from the isolation process by using a system of two immiscible liquids in the fractionation process—both as a stationary and mobile phase. These features provide small sample loss of the injected sample and high repeatability of the results. The developed method may be subject to upscaling, which is important to obtain high-purity compounds for biological research [12,13].

The sufficient amount of MGN isolated from the roots of *Berberis cretica* by counter-current chromatography enabled testing this compound for antitumor activity on cell lines of many types of neoplasms. From the initially tested seven cancer cell lines (A549, NCI-H1299, MCF7, MDA-MB-468, T98G, TE671, and HeLa) derived from five different tumors (lung, breast, glioblastoma, rhabdomyosarcoma, cervix), the authors selected four cell lines (NCI-H1299, MDA-MB-468, T98G, TE671) as the most promissing for a detailed investigation, where the IC_50_ of MGN was the lowest. Interestingly, these cell lines are derived from cancers that are difficult to treat and are characterized by very high proliferative potential. NCI-H1299 is a very aggressive non-small lung cancer cell line derived from the metastatic site (lymph node). The cells present a homozygous partial deletion of the p53 protein. NCI-H1299 cells show an aggressive mesenchymal phenotype with high vimentin expression [14]. MDA-MB-468 is a basal A triple negative breast cancer (TNBC) cell line, which does not express the estrogen receptor (ER), which limits the use of tamoxifen in chemotherapy. Moreover, the MDA-MB-468 cell line is characterized by numerous mutations in the p53 gene as well as has genetic amplification of the EGFR gene and very high expression of the EGFR protein [15]. The T98G cell line derived from a human glioblastoma multiform (GBM) tumor. GBM is one of the most malignant types of central nervous system tumors. Despite the latest radiative and chemical therapeutic approaches, it is still scarcely sensitive to these types of treatment and is generally considered an incurable cancer [16,17]. The TE671 cell line derived from rhabdomyosarcoma, which originates from mesenchymal stem cells, develops malignant tumors located in the head and neck or genitourinary system, causing very aggressive disease and clinical complications. Patients diagnosed with rhabdomyosarcoma have a poor prognosis, which is caused by late diagnosis, local recurrence, and metastasis [18]. Moreover, rhabdomyosarcoma is the most frequent childhood sarcoma of soft tissues [19]. The studies on the cellular and molecular mechanisms of action in the selected and above described cancer types—NCI-H1299 lung, MDA-MB-468 breast, T98G glioma and TE671 rhabdomyosarcoma cancer cells—is still missing in the scientific literature.

## 2. Materials and Methods

### 2.1. Reagents

Gradient grade solvents (methanol, ethyl acetate, butanol, 96% ethanol) that were used for the purification of MGN were purchased from Avantor Performance Materials (Gliwice, Poland). HPLC-MS purity solvents (water, acetonitrile, methanol, formic acid) for the extract’s profiling and purity check of the isolates were obtained from Merck (Darmstadt, Germany). Syringe nylon 0.22 µm membrane filters for the sample preparation were manufactured by Millex and were obtained from Merck (Darmstadt, Germany).

### 2.2. Plant Material

The dried roots of *Berberis cretica* used for the isolation of MGN were obtained from the Herbarium of the Department of Pharmacognosy and Natural Compounds Chemistry at University of Athens, Greece. The plant material was collected from Rouvas forest in Nidha Plateau, Crete in September 2010 and was authenticated by dr. Eleftherios Kalpoutzakis and prof. Alexios Leandros Skaltsounis from the same department. The obtained roots were finely cut, dried in the shade, ground, and their sample is deposited in the Chair and Department of Pharmacognosy of the Medical University of Lublin by the manuscript authors.

### 2.3. Extraction

First, 20 g of pulverized Cretan barberry root was transferred to a 33 mL stainless steel vessel and extracted in methanol by a pressurized liquid extractor ASE 100 (Dionex, Sunnyvale, CA, USA) according to the methodology previously published by the authors, with some modifications [11]. The following conditions were applied: extraction temperature: 80 °C, extraction time: 10 min, the number of extraction cycles: 3, purge time: 80 s, purge volume: 50%. During the extraction process, the pressure in the system was maintained at ca. 95 bar. The resulting extracts were joined and evaporated under reduced pressure at 45 °C to dryness yielding 2.7 g. The dry residue was used for the studies on Berberis cretica composition and isolation of MGN.

### 2.4. Qualitative Analysis of Berberis cretica Root Extract by HPLC-ESI-Q-TOF-MS

The qualitative analysis of the obtained extract and counter-current partition chromatography (CPC) fractions was performed by high-performance liquid chromatograph (HPLC) coupled with a high-resolution mass spectrometer: Q-TOF-MS by Agilent Technologies (Santa Clara, CA, USA) according to the previously published methodology with some modifications [20]. The high accuracy of mass measurement together with the studies on the molecules’ fragmentation patterns provided sufficient information for the identification of the major constituents of the extract. The obtained data were compared with those present in the scientific litrature and in open access databases, such as METLIN.

An HP1200 Series chromatograph applied in the studies was equipped in a binary pump, a degasser, and an autosampler, a column thermostat, a UV-PDA (Ultra Violet-Photo Diode Array) detector, and a Q-TOF mass spectrometer (6500 Series) with an electrospray ionization source (ESI). The chromatographic analysis was performed at 25 °C, within 22 min on a Zorbax Eclipse Plus stable bond RP-18 (150 × 2.1, 3.5 µm) chromatographic column (Agilent Technologies, Santa Clara, CA, USA) with a 5 min long post time. The injection volume was set at 20 µL and the gradient of 0.1% formic acid in acetonitrile (solvent A) in the 0.1% aqueous solution of formic acid was as follows: 0 min 10% A, 10–12 min 40% A, 17 min 95% A, 20 min 10% A. All samples were filtred through 0.22 µm nylon syringe filters before the injection on a chromatographic column.

The mass spectrometer was operated in both positive and negative ionization modes; however, the presence of alkaloids was recorded in the latter. The analyses were performed on a freshly tuned instrument within the m/z range of 40–1000 Da, under the following conditions: capillary voltage 3500 V, drying gas and sheath gas temperatures of 325 and 350 °C, gas flows of 12 L/min, fragmentation voltage of 140 and 200 V, the collision (CID) energies of 20 and 40 V, skimmer voltage of 65 V. The MS/MS spectra were collected for the two most abundant signals that were later excluded for the following 0.2 min to enable the fragmentation of less intensive signals. The Mass Hunter Workstation Software (v. B.08.00) was used for the acquisition and analysis of the recorded data.

### 2.5. Isolation of MGN from the Total Extract by Counter-Current Partition Chromatography (CPC)

The isolation of MGN from *B. cretica* methanolic root extract was performed on a counter-current partition chromatograph, CPC (Armen SCPC-250-L, Saint Ave, France), equipped with a 250 mL column, a UV detector, and a fraction collector. To achieve an effective purification of MGN, several biphasic solvent systems composed of ethyl acetate, butanol, and water in different ratios were studied. The following biphasic system—ethyl acetate/butanol/water (0.6:1.5:3 *v*/*v*/*v*)—showed the appropriate distribution of the target compounds and thus was selected for the isolation of the alkaloid [21]. The study on the partition coefficient values of the major constituents of the extract revealed a better distribution of MGN in aqueous phase, leading us to choose the descending mode for the CPC analysis. First, the slowly rotating column (500 rpm) was filled with 300 mL of the upper phase pumped with the flow rate of 20 mL/min. After the injection of the sample (300 mg of the extract in 6 mL of an equal mixture of the upper and lower phases), the elution was performed with the lower phase as the mobile phase, setting the rotation speed and flow rate at 1700 rpm and 6 mL/min, respectively. Then, 32 mL fractions were collected through the separation process. After 75 min, the analysis was carried out in the extrusion mode using the stationary phase as the mobile phase, still in the descending mode, for the following 40 min to empty the column. The composition of the collected fractions was determined by HPLC-ESI-Q-TOF-MS according to the above-described methodology. For this purpose, 1 mL of each collected fraction was evaporated to dryness using an Eppendorff Concentrator Plus (Eppendorff, Hamburg, Germany), re-dissolved in methanol, and filtered through a syringe filter prior to the chromatographic analysis.

### 2.6. Cell Lines

A549 (ATCC^®^ CCL185™) and NCI-H1299 (ATCC^®^ CRL-5803™) human lung cancer, MCF7 (ATCC^®^ HTB-22™) and MDA-MB-468 (ATCC^®^ HTB-132™) human breast cancer, T98G (ATCC^®^ CRL-1690™) human glioblastoma, TE671 (ATCC^®^ HTB-139™) human rhabdomyosarcoma, and HeLa (ATCC^®^ CCL-2™) human cervix cancer cells were obtained from the American Type Culture Collection (ATCC) (Manassas, VA, USA). Normal human primary fibroblast culture (HSF) was obtained by the outgrowth technique from skin explants of a young person, using a method routinely ongoing in the Biochemistry and Molecular Laboratory Laboratory at the Medical University of Lublin, Poland (Local Ethical Committee permission No KE0254/298/2015). A549, MCF7, MDA-MB-468, T98G, TE761, and HeLa cell lines were grown in Dulbecco’s Modified Eagle Medium/Nutrient Mixture F-12 (DMEM/F12) culture medium (Sigma, St. Louis, MO, USA), NCI-H1299 and HSF cells were maintained in RPMI1640 (Sigma) culture medium supplemented with 10% Fetal bovine serum (FBS) (Sigma, St. Louis, MO, USA), penicillin (100 IU/mL) (Sigma, St. Louis, MO, USA), and streptomycin (100 μg/mL) (Sigma, St. Louis, MO, USA) in a humidified atmosphere with 5% CO_2_ at 37 °C.

### 2.7. Cell Viability Assay

A549, NCI-H1299, MCF7, MDA-MB-468, T98G, TE671, and HeLa cancer cells (1 × 10^4^ cells/mL) [1] were plated on 96-well microplates. HSF normal cells were grown at a density of 1 × 10^5^ cells/mL. The cells were incubated in the presence of MGN (0.1–2 mg/mL) for 96 h. Then, the cells were incubated with the MTT (3-(4,5-dimethylthiazol-2-yl)-2,5-diphenyltetrazolium bromide) solution (5 mg/mL, Sigma) for 3 h. During this time, MTT was metabolized by living cells to purple formazan crystals, which were later solubilized in SDS buffer (10% SDS in 0.01N HCl). The optical density of the final product was measured with the use of an Infinite M200 Pro microplate reader (Tecan, Männedorf, Switzerland) at 570 nm.

### 2.8. Cell Proliferation—ELISA BrDU Assay

NCI-H1299, MDA-MB-468, T98G, and TE671 cells were placed on 96-well plates (Nunc, Roskilde, Denmark) at a density of 1 × 10^4^ cells/mL. After 24 h, cells were treated with 0.01–1 mg/mL of MGN for 48 h. DNA synthesis in proliferating cells was evaluated by measurement of 5-bromo-2′-deoxyuridine (BrdU) incorporation using a Cell Proliferation ELISA, 11647229001 BrdU kit (Roche, Basel, Switzerland). Absorbance was measured with the use of an Infinite M200 Pro microplate reader (Tecan, Männedorf, Switzerland) at 450 nm.

### 2.9. Assessment of Apoptosis

NCI-H1299, MDA-MB-468, T98G, and TE671 cancer cells were placed on 6-well plates (Nunc) at a density of 1 x 10^5^/mL, treated with MGN (1–10 mg/mL) for 48 h, harvested and washed with phosphate buffered saline (PBS). After that, cancer cells were fixed and permeabilized with the cytofix/cytoperm solution according to the manufacturer’s instruction of 550,914 PE Active Caspase-3 Apoptosis Kit (BD Pharmingen, CA, USA). Finally, cells were washed in the perm/wash buffer prior to intracellular staining with phycoerythrin (PE)-conjugated anti-active caspase-3 monoclonal rabbit antibodies. Labeled cells were analyzed to quantitatively assess the caspase-3 activity by flow cytometer FACSCalibur (Becton Dickinson, San Jose, CA, USA) operating with CellQuest software (Becton Dickinson).

### 2.10. Cell Cycle Analysis

NCI-H1299, MDA-MB-468, T98G, and TE671 cancer cells were placed on 6-well plates (Nunc) at a density of 1 × 10^5^/mL, treated with MGN (1–10 mg/mL), and then fixed in 80% ice-cold ethanol at −20 °C for 24 h. Next, the cells were stained with 550,825 propidium iodide (PI) utilizing the PI/RNase staining buffer (BD Biosciences, San Jose, CA, USA) according to the manufacturer’s protocol. The FACS Calibur TM flow cytometer (BD Biosciences, San Jose, CA, USA) was used for experiments. The cells were acquired and gated by using dot plot fluorescence (FL)-2 width (x) versus FL-2 area (y)-gate to exclude aggregates and analyzed in histograms displaying FL-2-area (yellow-orange fluorescence—585 nm). The acquisition rate was at least 60 events per second in low acquisition mode, and at least 10,000 events were measured. Cell cycle analysis was performed using flow cytometry analyzing software—WinMDI 2.9 for Windows (source: facs.scripps.edu/software.html) and Cylchred Version 1.0. 2 for Windows (source: University of Wales).

### 2.11. Statistical Analysis

GraphPad Prism 6 Software was used for statistical analysis. The calculations were done by one-way analysis of variance (ANOVA) test for multiple comparisons followed by Tukey’s significance test. Data are expressed as the mean standard error (SEM) (* *p* < 0.05, ** *p* < 0.01, *** *p* < 0.001). IC_50_ was calculated using computerized linear regression analysis of quantal log dose-probit functions according to the method of Litchfield and Wilcoxon [22].

## 3. Results

### 3.1. The Qualitative Composition of the Root Extract from Berberis cretica

**Figure 1:**
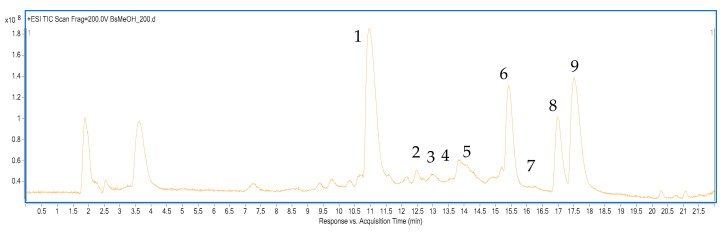
The total ion chromatogram of the methanolic extract from *Berberis cretica* roots.

The HPLC-ESI-Q-TOF-MS conditions applied in the study provided good separation of *Berberis cretica* metabolites and confirmed the presence of several alkaloids in its root MeOH extract (Figure 1). Among the major components from this group, MGN, jatrorhizine, palmatine, and berberine were tentatively identified (see Table 1) based on the accurate mass measurements, fragmentation patterns, and the published scientific literature. In addition, other minor constituents were found in the tested sample. Among them, coridynemethine, berbamine, berberrubine, obaberine or its isomer, armepavine, and berbamine were traced. All components represent protoberberine, aporphine, and benzylisoquinoline types of isoquinoline alkaloids.

### 3.2. The Fractionation of the Extract by CPC Chromatography

The applied CPC settings and the composition of the biphaasic solvent system provided a fast and effective purification of MGN from the root extract of *Berberis cretica*. The purification was possible thanks to the high selectivity of the prepared solvent mixture that was proved by distant partition coefficient values (K) of the major peaks. The K values that resemble the actual affnity of the extract’s components to the solvent system were calculated as the peak area of a given compound in the stationary (organic) phase divided by its peak area in the mobile (aqueous) phase [12]. The obtained values for the major alkaloids were as follows: for MGN, 0.6; for the remaining compounds, 1.8, 2.3, 2.8, and 3.4. In the analyzed conditions, MGN was the only major alkaloid characterized by a better solubility in the aqueous phase.

**Figure 2:**
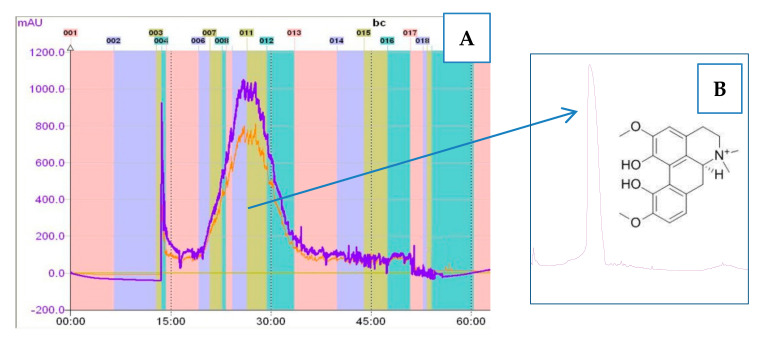
The part of counter-current partition chromatography (CPC)-UV chromatogram (at 290 nm) of the *Berberis cretica* root extract that show the elution of magnoflorine (MGN) (**A**) and the total ion chromatogram (TIC) of the isolated MGN (**B**).

During the chromatographic separation, MGN was eluted as early as 25 min after the injection and was collected over a period of 10 min (Figure 2A). The purity of the isolated alkaloid (Figure 2B) was calculated as 95.7% by the Mass Hunter Work Station program. Then, 18 mg of magnoflorine was obtained from the injected 300 mg of the extract in one run. The effectiveness of purification was related to the fact that among the identified alkaloids, MGN was the only compound that belonged to the group of aporphines, and due to a clear difference in the chemical structure, it could be more effectively separated from protoberberines by a simple biphasic solvent system. The fragmentation pattern of MGN was in accordance with the previously described data. The MS/MS spectrum showed clear *m*/*z* signals from the subsequent deattachments of the methyl and methoxyl groups from the molecular ion according to the previously published work of Tian and colleagues [23].

### 3.3. MGN Significantly Reduces the Viability of Cancer Cells

**Figure 3:**
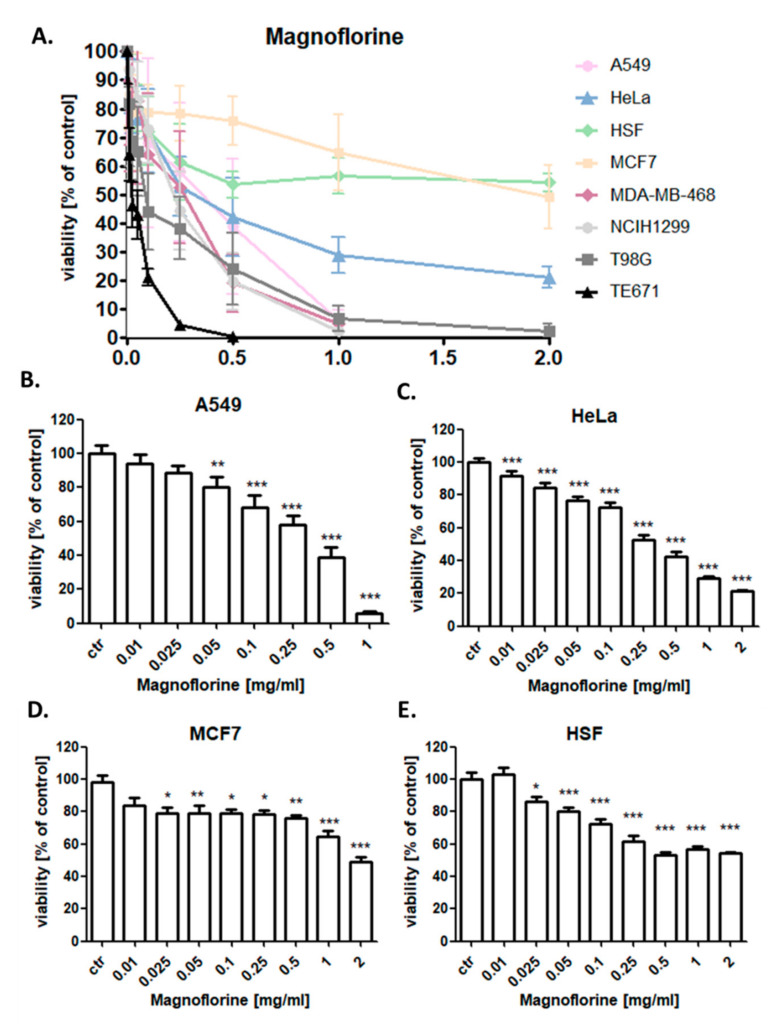
Effect of magnoflorine (MGN) on viability of human lung cancer (A549 (**A**,**B**) and H1299 (**A**) [1]), breast cancer (MCF7 (**A**,**D**) and MDA-MB-468 (**A**) [1]), cervix cancer (HeLa) (**A**,**C**), glioma (T98G (**A**) [1]), rhabdomyosarcoma (TE671 (**A**) [1]), and human skin fibroblast (HSF (**A**,**E**)) cells. The cancer cells were exposed to either culture medium alone (control) or magnoflorine (0.01–2 mg/mL) for 96 h. Normalized cell viability measured by the methylthiazolyldiphenyl-tetrazolium bromide (MTT) assay is presented as mean ± SD at each concentration. The differences between groups were evaluated using the one-way ANOVA; Tukey’s post-hoc test. *** *p* < 0.001, ** *p* < 0.01, * *p* < 0.05; n = 18 per concentration from three independent experiments.

The anti-proliferative activity of MGN (0.1–2 mg/mL) for 96 h was determined in lung (A549), breast (MCF7), and cervix cancer (HeLa) cell lines using the MTT assay in order to establish the IC_50_ value for MGN (Table 2). The influence of MGN (0.1–2 mg/mL) on the viability of NCI-H1299 lung cancer, MDA-MB-468 breast cancer, T98G glioblastoma, and TE671 rhabdomyosarcoma cells was investigated previously [1]. In the studies, the dose-dependent growth inhibition effect of MGN was evident in all analyzed cancer cell lines. As shown in Figure 1, various cancer cell lines displayed differential responses when treated with MGN. TE671 cells [1] were the most sensitive and MCF7 cells were the least sensitive to MGN treatment among all analyzed cancer cell lines with IC_50_ = 22.83 µg/mL [1] and 1960.8 µg/mL, respectively. MDA-MB-468 breast cancer cells (IC_50_ = 187.32 µg/mL) [1] were much more sensitive to MGN than MCF7 cells. Moreover, the inhibitory effect of MGN on the growth of studied lung cancer cell lines was more relevant on NCI-H1299 cells (IC_50_ = 189.65 µg/mL) [1] than on the A549 cell line (IC_50_ = 296.7 µg/mL). The design or discovery of new anti-cancer active agents showing selectively toxicity to tumor cells without affecting normal cells is the main goal of anti-cancer therapy. Consistent with this concept, the cytotoxic activity of MGN against HSF normal cells in MTT assay was analyzed. No very strong cytotoxic effect of MGN was observed on HSF normal cells (Figure 3) in the concentration range of 10 to 2000 µg/mL, which did not allow calculating the IC_50_ value for this cell line. TE671 rhabdomyosarcoma, T98G glioblastoma, MDA-MB-468 breast cancer, and NCI-H1299 larynx cancer cells showed the best response to MGN treatment (Table 2) [1], and thus they were further used to explore a possible underlying MGN-mediated mechanism of anti-cancer action on the cellular level.

### 3.4. MGN Slightly Reduces the Proliferation of NCI-H1299, MDA-MB-468, T98G, and TE671 Cancer Cells

**Figure 4:**
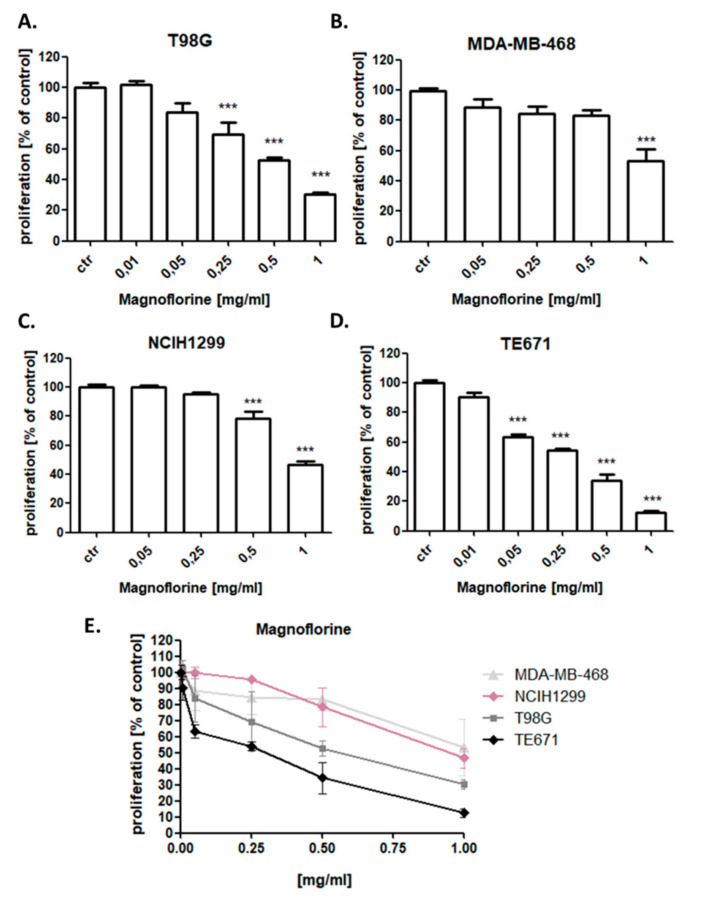
Effect of magnoflorine (MGN) on the proliferation of T98G glioma (**A**,**E**), MDA-MB-468 breast (**B**,**E**), NCIH1299 lung (**C**,**E**), and TE671 rhabdomyosarcoma (**D**,**E**) cancer cells in 5-bromo-2ʹ-deoxyuridine (BrdU) assay. Cancer cells were incubated for 48 h alone (control) or in the presence of magnoflorine (0.01–1 mg/mL). The differences between groups were evaluated using the one-way ANOVA; Tukey’s post-hoc test. *** *p* < 0.001. Results from three independent experiments were presented as mean ± SD of the mean.

The effect of MGN on the cancer cells proliferation was attributed to decreased cell division, as determined by the decreased incorporation of BrdU. NCI-H1299, MDA-MB-468, T98G, and TE671 cancer cells were exposed to either culture medium (control) or MGN in the concentration range from 0.01 to 1 mg/mL. MGN slightly reduced the proliferation of all analyzed cancer cells in a dose-dependent manner after 48 h of incubation with MGN. The most statistically significant anti-proliferative effect of MGN was observed in TE671 rhabdomyosarcoma cell line, whereas MDA-MB-468 cells were the most resistant for MGN treatment among all analyzed cancer cell lines (Figure 4).

### 3.5. MGN Induces Apoptosis in NCI-H1299, MDA-MB-468, T98G, and TE671 Cancer Cells

**Figure 5:**
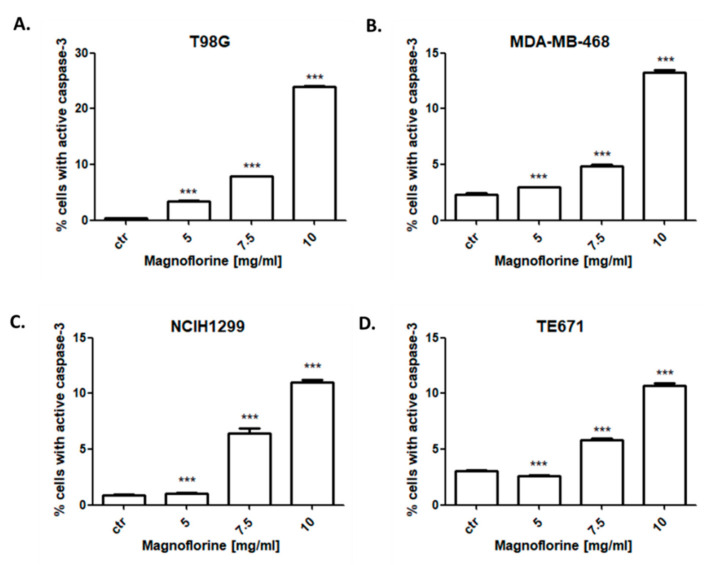
Effects of magnoflorine (MGN) on caspase-3 activation in T98G glioma (**A**), MDA-MB-468 breast (**B**), NCIH1299 lung (**C**), and TE671 rhabdomyosarcoma (**D**) cancer cells. Cancer cell lines were cultivated for 48 h with different doses of MGN (5–10 mg/mL) and analyzed by flow cytometry. The values present the percentage of the cells with active caspase-3. The results are depicted as means ± SD, n = 9 from three separate experiments. Statistical analysis was performed using one-way ANOVA test. *** *p* < 0.001.

**Figure 6:**
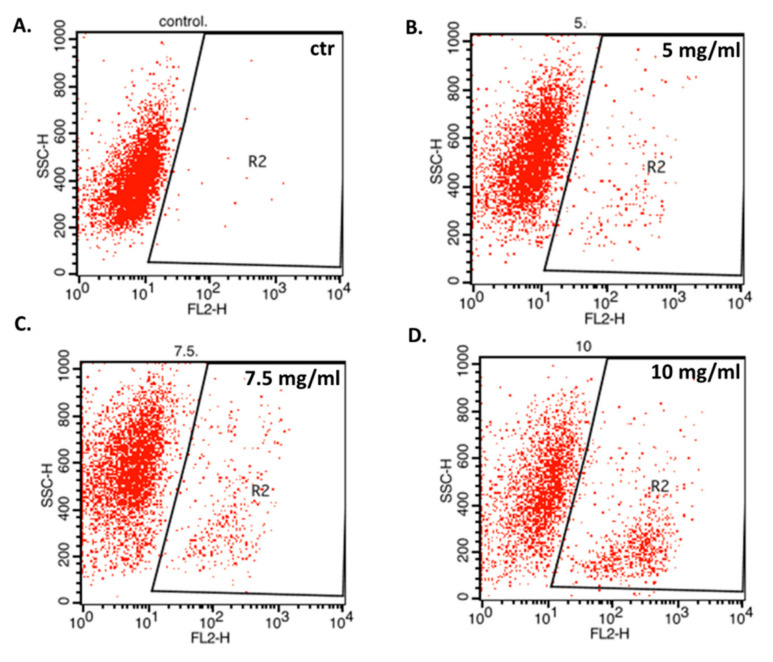
Representative dot plots of the flow cytometry analysis of T98G glioma cells after 48 h treatment with medium (ctr) (**A**) or magnoflorine (5 mg/mL) (**B**), 7.5 mg/mL (**C**), and 10 mg/mL (**D**); n = 9 per concentration from three independent experiments. R2-cells with active caspase-3 (apoptotic cells).

In the next experiments, correlation of the anti-proliferative effect of MGN with apoptosis induction was determined. All cancer cell lines were treated with 5–10 mg/mL MGN for 48 h. The study showed that MGN slightly triggered apoptotic cell death in NCI-H1299, MDA-MB-468, T98G, and TE671 cells in a dose-dependent manner (Figure 5). The most evident increase in the number of active caspase-3-positive cells after MGN treatment was observed in T98G glioma cells (Figure 6). As shown in Figure 3, the percentage of apoptotic T98G cells after treatment with 10 mg/mL MGN was found to be 24.02%; *** *p* < 0.05 (Table 3). MGN slightly increased the percentage of apoptotic cells in TE671 (10.71%), NCI-H1299 (10.98%), and MDA-MB-468 (13.26%) cancer cells after MGN treatment (10 mg/mL) (Table 4).

### 3.6. MGN Influences Cell Cycle Progression in the S/G2 Phases in NCI-H1299, MDA-MB-468, and T98G Cancer Cell Lines

**Figure 7:**
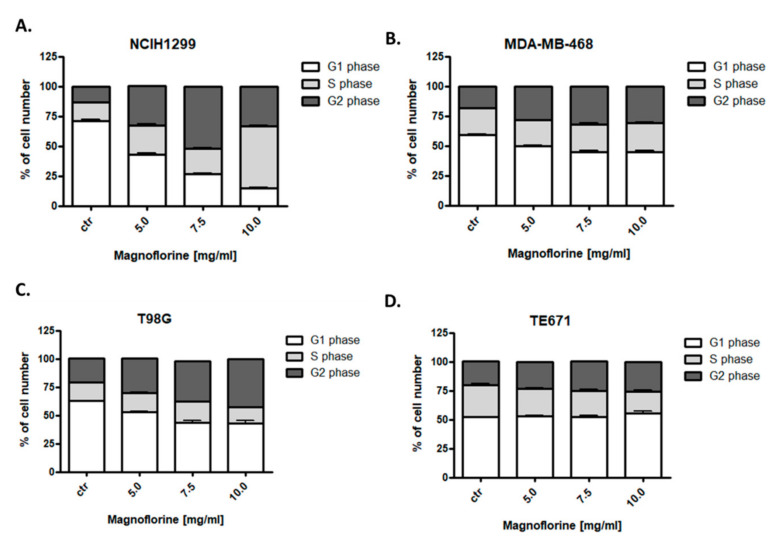
Effects of magnoflorine (MGN) on the cell cycle progression in NCIH1299 lung (**A**), MDA-MB-468 breast (**B**), T98G glioma (**C**), and TE671 rhabdomyosarcoma (**D**) cancer cells. Cancer cell lines were cultivated for 48 h with different doses of magnoflorine (5–10 mg/mL) and analyzed by flow cytometry. The results are depicted as means ± SD, n = 9 from three separate experiments.

Since an inhibition of cell proliferation resulted from decreased cell division, cell cycle analysis by means of flow cytometry were performed. Effects of MGN treatment (5–10 mg/mL) on the cell cycle progression were examined in four (NCI-H1299, MDA-MB-468, T98G, and TE671) cancer cell lines. Fluorescence-activated cell sorting (FACS) analysis of PI-stained cells indicated that the incubation of NCI-H1299, MDA-MB-468, and T98G cancer cells with MGN for 48 h led to an accumulation of cells in the S/G2 phases of the cell cycle in dose-dependent manner (Figure 7) (Table 4). The strongest effect was observed in the NCI-H1299 lung cancer cell line (Figure 7). An incubation of TE671 cells with MGN resulted in a cell cycle inhibition pattern similar to the control. Despite the fact that this cell line was the most sensitive to MGN (IC_50_ = 22.83 µg/mL), no changes in the cell cycle were detected (Figure 7), which may indicate that the mechanism of action of this compound in TE671 cells is completely different than in other cell lines, which requires further research. Detailed data are shown in Table 4.

## 4. Discussion

Despite significant advances in cancer therapy, patient survival rates are still low. The inadequate efficacy of standard anti-cancer therapies and their serious side effects led to a renewed interest in natural medicine to identify bioactive compounds of plant origin with promising chemotherapeutic properties [30]. MGN, a quaternary aporphine alkaloid widely distributed in many species of plants, seems to be a promising active agent with potential anti-cancer properties. Despite an increasing number of studies on this compound, which confirm its activity, the availability of analytical techniques that could isolate it from plant material is still negligible. Modern pharmacognosy and phytotherapy necessitate the isolation of single compounds from a rich mixture of chemical structures such as plant extracts to better predict the possible presence of side effects or interactions with other drugs. Among the several separation techniques that offer a preparative scale of operation, counter-current chromatography is certainly worth mentioning.

Counter-current partition chromatography (CPC) is a modern separation technique that enables the isolation of individual components from mixtures of organic compounds, e.g., from plant extracts, using a system of two immiscible liquids (liquid–liquid partition chromatography), one of which is retained on a rotating column (stationary phase), and the second washes it (mobile phase). The actual separation process on a rotating column depends on the differences in the affinity of individual components toward the stationary or mobile phase. A large variety of solvents that can be used to formulate the separation mixture makes this technique versatile and suitable for the fractionation of chemically differentiated metabolites with no risk of sample loss due to their adsorption on solid phases. In addition, the separation process in counter-current chromatography takes place with little consumption of basic-purity solvents and the transfer of analytical conditions to an industrial or preparative scale is uncomplicated, which is helpful in isolating natural compounds for biological studies where a higher quantity is necessary [31].

The herein described way of MGN purification from the dried root extract of Cretan barberry using CPC chromatography enabled the isolation of the alkaloid on a semi-preparative scale, in a fast and efficient manner, with the purity exceeding 95.7%. Thanks to the elimination of a solid adsorbent, the authors managed to exclude the tailing process of other alkaloids present in the extract, contaminating MGN, as was the case with classic isolations (e.g., contamination with berberine). Moreover, the developed isolation methodology offers the upscaling possibilities expressed with an enlarged injection volume and concentration of crude extracts still with no need for any initial sample preparation. In view of the above, this methodology seems to be universal and will certainly enable high-throughut spearation of MGN. The here presented methodology is a modification of the previously published protocol. The application of a higher rotation speed of the column provided higher purity of the isolate, and that is why no further purification steps are necessary [21]. The scientific literature lists only a few examples of counter-current chromatography-based purification of this alkaloid. The majority of the trials come from the authors of the manuscript and denote the isolation of MGN from *Berberis spp* [21], whereas other researchers isolated this compound from *Zanthoxylum alianthoides* [32] by means of pH-zone refining chromatography. In comparison with [33,34], the herein described protocol does not include the introduction of the pH-zone refining mode. As a consequence, the proposed composition of the biphasic solvent system with no modifiers added allowed a quick elution of MGN out of the column. In comparison with [32], MGN was leaving the column at the very beginning of the method, which provided it with high purity and short analysis time.

Having studied a variety of plant extracts from the representatives of *Berberis*, *Argemone*, *Papaver*, and *Eschscholzia* genders [11], the authors selected the roots from *Berberis cretica* as the most rich source of MGN. Additionally, the composition of the extract seemed quite poor in other metabolites, and that is why this plant source seems to be a promising one for the future isolation of this aporphine alkaloid for pharmacological treatment.

It has been demonstrated that MGN shows many potential therapeutic properties including antifungal [7], neuropsychopharmacological [2], anti-anxiety [3,4], anti-inflammatory [3], immunomodulatory [5], or antioxidant [6] activities. Several studies evidenced also the anti-cancer activity of MGN [1,8,9]. The mechanism of action of MGN has been studied, either individually or in combination with other cytostatics, in several malignant types, including gastric [9], breast (MGN+doxorubicin (DOX)) [8] or osteosarcoma (MGN+cisplatin (CDDP)) [33]. In our previous study, the types of pharmacological interactions between MGN and CDDP in breast, lung, glioma, and rhabdomyosarcoma cancer cells using isobolographic method were assesed [1]. The ability of MGN to inhibit cancer cell growth was demonstrated against several malignant cell lines, including A549 and NCI-H1299 human lung cancer, MCF7 and MDA-MB-468 human breast cancer, T98G human glioblastoma, TE671 human rhabdomyosarcoma, and HeLa human cervix cancer cells. TE671 rhabdomyosarcoma, T98G glioblastoma, MDA-MB-468 breast cancer, and NCI-H1299 larynx cancer cells showed the best response to MGN treatment [1], and thus, these cell lines were further used to explore a possible underlying MGN-mediated mechanism of anti-cancer action on the cellular level. Interestingly, in the previous studies, no very strong cytotoxic effect of MGN was observed on HSF normal cells. It has been found that MGN decreased the viability of cancer cells by promoting apoptosis, following or concurrent with cell cycle arrest at the S/G2 phases. However, cell cycle inhibition or induction of apoptosis occurred at very high doses of MGN (5–10 mg/mL) used. It has been revealed that MGN has a very low bioavailability index and high absorption and elimination rates [3].

It has been shown that MGN inhibits the viability, invasion, and epithelial–mesenchymal transition (EMT) of osteosarcoma cells in a dose-dependent manner. MGN significantly suppressed NF-κB (nuclear factor kappa-light-chain-enhancer of activated B cells) activation and HMGB1 (high mobility group box 1 protein) expression, but it upregulated miR-410-3p level. The miR-410-3p mimic inhibited the EMT of osteosarcoma cells, which was restored by an upregulation of HMGB1 [33]. Another group revealed that MGN suppresses the proliferation of gastric cancer cells but showed no influence on the normal gastric cells. It has been demonstrated that MGN induces autophagy in gastric cancer cells by the up-regulation of LC3B-II expression and reduction of the formation of autophagosome. MGN-triggered autophagic cell death was regulated by reactive oxygen species (ROS)-induced suppression of serine/threonine-protein kinases (AKT) signaling. Similarly to the achieved results, MGN treatment leads to apoptosis through enhancing cleaved caspase-3. Moreover, an up-regulated expression of p21 and p27 proteins, as well as down-regulated expression of cyclin-A and cyclin-B1, was observed after MGN treatment in gastric cancer cells, contributing to the S/G2 cell cycle arrest [9]. MGN obtained from a fractionation of the methanol extract from *Magnolia grandiflora* leaves inhibited the viability of the U251 brain cancer, Hela cervix cancer, and HEPG2 hepatocellular carcinoma cell lines. It has been demonstrated that IC_50_ of MGN against HEPG2 cells (0.4 mg/mL) was only two times higher than IC_50_ of doxorubicin (DOX) [34]. In turn, another research group demonstrated that MGN isolated from the fruit of *Ziziphus jujube* showed a very weak, statistically insignificant cytotoxic effect against A549 lung cancer, MCF7 breast cancer, HT-29 colon cancer, and HepG2 hepatocellular carcinoma cell lines [35].

Since MGN is not highly toxic to normal cells but unfortunately exhibits anti-tumor activity at high concentrations, it is worth considering the use of this compound in combination therapy with standard cytostatics. In the context of anti-cancer therapy, causing often severe toxic side effects, the simultaneous use of MGN and standard cytostatics seems to have a beneficial influence, potentially ameliorating some conventional drug-induced side effects. In the previous manuscript, it has been shown have shown that MGN in combination with CDDP at a fixed ratio of 1:1 augmented their anti-cancer activity and yielded synergistic or additive pharmacological interactions by means of the isobolographic method; therefore, combined therapy using these two active agents can be a promising chemotherapy regimen in the treatment of some types of breast, lung, rhabdomyosarcoma, and glioblastoma cancers [1]. It has been demonstrated that MGN inhibits the malignant phenotypes and increases the CDDP sensitivity of osteosarcoma cells via modulating the miR-410-3p/HMGB1/NF-κB pathway [33]. Another group revealed that MGN improves sensitivity to DOX via inducing apoptosis and autophagy through AKT/mTOR (mammalian target of rapamycin) and p38 signaling pathways in breast cancer cells. MGN strongly promoted a DOX-induced anti-proliferative effect in breast cancer cells but not in human normal cells. DOX-triggered DNA damage was accelerated by combined treatment with MGN in breast cancer cells. Additionally, DOX-induced cell distribution in the G2/M phase was significantly elevated after co-treatment with MGN. Combined treatment MGN with DOX also promoted the p38 mitogen-activated protein kinase (MAPK) pathway and significantly inhibited the activation of phosphatidylinositol 3-kinase/protein kinase B/mammalian target of rapamycin (PI3K/AKT/mTOR) signaling [8].

Magnoflorine was found to be better absorbed than other alkaloids from the same group, such as palmatine, berberrubine, or epiberberine. Chen et al. demonstrated that the bioavailability of MGN is 10-fold greater than of the other alkaloids from the same group and can be compared to berberine, which has been already well studied and proved to be an anti-cancer agent [36]. These data are promising enough to assume that the bioavailability of MGN is sufficient to achieve the concentrations necessary to induce the anticancer activity that is indicated in our work. Xue et al. [37] proved that MGN in the form of a single compound is absorbed in the most efficient manner in the jejenum and ileum parts of small intestine (1.97 ± 0.25 and 1.31 ± 0.23 µg/cm^3^, respectively). In addition, it has been shown that the combination of phospholipids and MGN increased its ability to penetrate the blood–brain barrier, which is very important in the treatment of gliomas (cell line T98G) [2].

All these results indicate that MGN might be a novel promising drug for the treatment of some types of cancers, especially in combination with other clinically available anti-cancer drugs. However, the mechanism of action of MGN, individually or in combination with other active agents, should be validated by functional in vitro and in vivo studies.

## 5. Conclusions

Counter-current partition chromatography is a new and effective separation technique that allows to obtain high purity MGN for biological research. MGN at high doses is active against neoplasms where the effectiveness of chemotherapy is low. The mechanism of action of MGN at the cellular level may be tumor dependent, which requires further investigation.

## Figures and Tables

**Table 1:** The list of tentatively identified alkaloids in the root extract from *Berberis cretica* by HPLC-ESI-Q-TOF-MS.

No	Ion (+/-)	Rt (min)	Molecular Formula	m/z Calculated	m/z Experimental	Delta (mmu)	RDB	MS/MS Fragments	Proposed Compound	Ref.
**1**	[M + H]^+^	10.9	C_20_H_23_NO_4_	342.1700	342.1704	−1.22	10	297, 282, 265, 237	**Magnoflorine**	[23]
**2**	[M + H]^+^	12.2	C_19_H_23_O_3_N	314.1750	314.1758	−0.2	8.5	269, 175	**Armepavine**	[24]
**3**	[M + H]^+^	13.0	C_21_H_25_NO_4_	356.1856	356.1865	−2.44	10	311, 279, 251	**Corydinemethine**	[25]
**4**	[M + H]^+^	13.5	C_37_H_40_N_2_O_6_	609.2959	609.2967	−1.29	19	578, 566, 401, 305	**Berbamine**	[26]
**5**	[M + H]^+^	14.0	C_38_H_42_N_2_O_6_	623.3116	623.3131	−2.47	19	592, 580, 312	**Obaberine/berbamunine**	[27]
**6**	[M + H]^+^	15.4	C_20_H_19_NO_4_	338.1385	338.1391	−1.23	12	323, 308, 294, 280	**Jatrorrhizine**	[28]
**7**	[M + H]^+^	16.2	C_19_H_15_NO_4_	322.1074	322.1078	−1.29	13	307, 292, 280	**Berberrubine**	[29]
**8**	[M + H]^+^	16.9	C_21_H_21_NO_4_	352.1543	352.1552	−2.46	12	337, 322, 308	**Palmatine**	[21]
**9**	[M + H]^+^	17.5	C_20_H_21_NO_4_	336.1230	336.1244	−4.07	13	321, 306, 292, 278, 262	**Berberine**	[21]

**Table 2:** Half-maximal inhibitory concentrations (IC_50_ ± SD) for the magnoflorine (MGN) for A549, NCI-H1299 [1] lung; MCF7, MDA-MB-468 [1] breast; T98G [1], glioma; TE671 [1], rhabdomyosarcoma; and HeLa cervix cancer cells calculated using computerized linear regression analysis of quantal log dose–probit functions according to the method of Litchfield and Wilcoxon [22].

Type of Cancer	Cell Line	IC_50_ (µg/mL)
Breast cancer	MDA-MB-468	187.32 ± 45.80
	MCF7	1960.80 ± 528.78
Lung cancer	NCI-H1299	189.65 ± 48.97
	A549	296.7 ± 51.23
Cervix cancer	HeLa	315.4 ± 62.18
Glioma	T98G	112.12 ± 48.06
Rhabdomyosarcoma	TE671	22.83 ± 8.65

**Table 3:** The percentage of T98G, MDA-MB-468, NCI-H1299, and TE671 cancer cells positive for active caspase-3 after treatment with MGN for 48 h. Statistical analysis was performed using one-way ANOVA test (*** *p* < 0.001).

Cell Line	Concentration [mg/mL]	Mean	SD	Statistical Significance
T98G	ctr	0.424	0.2411	
	5	3.435	0.3552	***
	7.5	7.890	0.1214	***
	10	24.02	0.2121	***
MDA-MB-468	ctr	2.297	0.2411	
	5	2.963	0.04933	***
	7.5	4.883	0.1850	***
	10	13.26	0.3816	***
NCI-H1299	ctr	0.900	0.1562	
	5	1.057	0.05508	***
	7.5	6.390	0.8861	***
	10	10.98	0.3736	***
TE671	ctr	3.070	0.07211	
	5	2.573	0.1343	***
	7.5	5.817	0.3137	***
	10	10.71	0.3396	***

**Table 4:** Cell cycle analysis of T98G, MDA-MB-468, NCI-H1299, and TE671 cancer cells after treatment with MGN for 48 h.

Cell Line	Concentration [mg/mL]	G1 Phase [%]	S Phase [%]	G2 Phase [%]
T98G	ctr	62.63	16.44	21.35
	5	53.2	16.85	30.13
	7.5	43.62	18.75	35.36
	10	43.11	14.23	42.45
MDA-MB-468	ctr	59.47	21.9	11.69
	5	50.03	21.63	27.88
	7.5	45.10	23.11	31.55
	10	44.56	24.52	30.86
NCI-H1299	ctr	71.02	15.35	13.45
	5	42.76	24.4	32.97
	7.5	26.89	20.99	52.06
	10	14.8	51.78	33.06
TE671	ctr	52.07	27.93	20.28
	5	53.02	23.41	23.18
	7.5	52.3	22.42	25.49
	10	55.53	18.93	25.31
